# Localization of breast cancer resistance protein (Bcrp) in endocrine organs and inhibition of its transport activity by steroid hormones

**DOI:** 10.1007/s00441-012-1417-5

**Published:** 2012-05-13

**Authors:** Anita C. A. Dankers, Fred C. G. J. Sweep, Jeanne C. L. M. Pertijs, Vivienne Verweij, Jeroen J. M. W. van den Heuvel, Jan B. Koenderink, Frans G. M. Russel, Rosalinde Masereeuw

**Affiliations:** 1 Department of Pharmacology and Toxicology (149), Nijmegen Centre for Molecular Life Sciences / Radboud University Nijmegen Medical Centre, P. O. Box 9101, 6500 HB Nijmegen, The Netherlands; 2 Department of Laboratory Medicine, Radboud University Nijmegen Medical Centre, Nijmegen, The Netherlands

**Keywords:** BCRP, ABCG2, Endocrine organs, Steroids, Transport inhibition

## Abstract

Breast cancer resistance protein (BCRP) is known for its protective function against the toxic effects of exogenous compounds. In addition to this, a role in the transport of endogenous compounds has been described. Since BCRP in the plasma membrane was shown to be regulated by sex steroids, we investigated the presence and possible role of BCRP in steroid hormone-producing organs. Therefore, the presence and localization of Bcrp was investigated in endocrine organs of wild-type mice. Furthermore, the interaction of various steroid hormones with human BCRP activity was studied. Quantitative PCR revealed *Bcrp* mRNA in the pituitary and adrenal glands, pancreas, ovary, testis and adipose tissue. Immunohistochemistry revealed the presence of Bcrp in the cortex of the adrenal gland and in plasma membranes of adipocytes. In the pituitary gland, pancreas, ovary and testis, Bcrp was mainly located in the capillaries. The interaction between BCRP and 12 steroid hormones was studied using membrane vesicles of HEK293-BCRP cells. Estradiol, testosterone, progesterone and androstenedione inhibited BCRP-mediated uptake of ^3^H-estrone sulphate (E_1_S) most potently, with calculated inhibitory constant (*K*i) values of 5.0 ± 0.2, 36 ± 14, 14.7 ± 1.3 and 217 ± 13 μM, respectively. BCRP function was attenuated non-competitively, which implies an allosteric inhibition of BCRP-mediated E_1_S transport by these steroids. In conclusion, localization of Bcrp in endocrine organs together with the efficient allosteric inhibition of the efflux pump by steroid hormones are suggestive for a role for BCRP in steroid hormone regulation.

## Introduction

Breast cancer resistance protein (BCRP; ABCG2) is a member of the ATP binding cassette (ABC) superfamily of transmembrane proteins involved in the transport of a variety of molecules against steep concentration gradients at the expense of ATP. BCRP is a half transporter that requires dimerization to become a fully active efflux pump and was originally discovered in a breast cancer cell line resistant to chemotherapeutics (Mao and Unadkat 2005; Doyle et al. 1998; Ross et al. 1999). Nowadays, BCRP is recognized as a xenobiotic transporter that plays a major role in multidrug resistance (Jonker et al. 2005; Mao and Unadkat 2005; Doyle and Ross 2003). The efflux pump is present in various tissues with a barrier function, including the placenta, prostate, small intestine, brain, colon, liver, mammary gland and kidney (Maliepaard et al. 2001; Doyle and Ross 2003; Bart et al. 2004; Fetsch et al. 2006; Huls et al. 2008). In addition to the protective role of BCRP against accumulating xenobiotic compounds, a role in the transport of endogenous compounds has been described, such as the transport of folic acid (Chen et al. 2003), heme (Krishnamurthy et al. 2004; Zhou et al. 2005; Suzinges-Mandon et al. 2010), urate (Woodward et al. 2009; Matsuo et al. 2009) and uremic toxins (Mutsaers et al. 2011). Moreover, BCRP transports conjugated steroids, such as dehydroepiandrosterone sulfate (DHEAS) (Lee et al. 2005), estrone sulfate (E_1_S) and estradiol glucuronide (E_2_17βG) (Chen et al. 2003; Imai et al. 2003; Suzuki et al. 2003).

The localization of BCRP in the plasma membrane is shown to be sex specific, which is suggested to be due to the suppressive effect of estradiol and the inductive effect of testosterone (Ee et al. 2004; Imai et al. 2005; Hartz et al. 2010; Tanaka et al. 2005). Also, progesterone has been shown to regulate BCRP transcription in cancer cell lines, including BeWo cells, a cell line derived from human placental choriocarcinoma (Wang et al. 2008). Moreover, a role for BCRP in transport of androgens has been suggested in prostate stem cells (Huss et al. 2005) and transport of estradiol was demonstrated in membrane vesicles from *Lactococcus lactis* containing functional human BCRP (Janvilisri et al. 2003), indicating that the efflux pump might also play a role in steroid action. In this respect, the presence of BCRP has been described for some hormone-producing organs (Fetsch et al. 2006; Langmann et al. 2003; Tanaka et al. 2005) but for some organs, only *BCRP* mRNA content or presence of the protein by western blot has been shown. In the present study, we investigated murine organs, including the pituitary and adrenal glands, pancreas, kidney, ovary, testis and adipose tissue, for the presence and localization of Bcrp. Furthermore, we evaluated the effects of several sex steroids on BCRP-mediated substrate transport using membrane vesicles of baculovirus-transduced HEK293 cells. Our results show the presence of the efflux pump in steroid-producing organs. In addition, the sex steroids estradiol, testosterone, progesterone and androstenedione inhibited BCRP function in a concentration-dependent manner.

## Materials and methods

### RNA isolation and quantitative PCR

All procedures involving animals were approved by the Animal Experimental Committee of the Radboud University Nijmegen Medical Centre. *Bcrp* mRNA was assessed in the adrenal gland, pituitary gland, epididymal fat pad, abdominal fat pad, pancreas, testis, ovary and kidney of Friend leukemia virus B (FVB) mice (Charles River Laboratories, Germany). Isolated organs were immediately snap-frozen in liquid nitrogen until further analysis. Fat pads, adrenal glands and pituitaries were homogenized using micropestles. Other organs were homogenized in frozen state using a Mikro-dismembrator U (Sartorius B. Braun Biotech, Melsungen, Germany). To avoid RNA degradation, the metal cylinders were washed with 0.5 M NaOH prior to use. Subsequently, total RNA was isolated using a NucleoSpin^®^ RNA II kit (Macherey-Nagel, Düren, Germany) according to the manufacturer’s instructions. Immediately, a reverse transcriptase reaction was performed with 250 ng RNA using random primers (Invitrogen, Breda, The Netherlands) and an Omniscript^®^ RT kit (Qiagen, Hilden, Germany), following the manufacturer’s recommendations. Synthesized cDNA was used for quantitative PCR, performed in a StepOnePlus™ Real-Time PCR system by means of the TaqMan® protocol (Applied Biosystems, Warrington, UK). *Bcrp* mRNA concentration was normalized to the mRNA concentration of the housekeeping gene *ß-actin*. The primer-probe sets were obtained from Applied Biosystems (*β-actin*: 4352933E; *Bcrp*: Mm00496364_m1).

### Immunohistochemistry

The localization of Bcrp was assessed by immunohistochemistry. Organs from Bcrp^−/−^ mice were used as negative controls. The knockout mice were kindly provided by Dr. A. Schinkel (Netherlands Cancer Institute, Amsterdam, The Netherlands) and were bred and housed at the Central Animal Laboratory of the Radboud University Nijmegen Medical Centre. The organs were fixed by cardiac perfusion. Briefly, the mice were anesthetized with a single, lethal i.p. injection of 100 mg/kg pentobarbital (Nembutal, 60 mg/ml). The hearts of anesthetized mice were exposed and the right atrium was clipped with surgical scissors. A 23-gauge needle was inserted into the left ventricle and phosphate buffered saline (PBS) containing heparin (300 ml/h) was administered. Subsequently, the mice were perfused with a 4 % w/v formaldehyde solution, freshly prepared from paraformaldehyde. The organs were removed and fixed in a 4 % w/v formaldehyde solution for 24 h and embedded in paraffin, except for the fat at pads, which were fixed in Bouin’s fixative immediately after the perfusion fixation. Sections of 5 μm were mounted on 3-aminopropyltriethoxysilane (APES)-coated slides and dried for 2 h at 57 °C. After deparaffinization with xylene and rehydration, the slides were heated in sodium citrate buffer (pH 6) at 100 °C for 15 min. Endogenous peroxidase was blocked with 1.5 % v/v H_2_O_2_ for 30 min. After blocking with nonimmune rabbit serum, the slides were incubated with primary antibody against mouse Bcrp (BXP-9, 1:20; Kamiya Biomedical, Seattle, WA, USA) overnight at 4 °C. The biotinylated secondary antibody (rabbit anti rat, 1:500; Acris Antibodies, Herford, Germany) was incubated for 30 min followed by a 30-min incubation with standard avidin-biotin complex (ABC; Brunschwig Chemie, Amsterdam, The Netherlands). Next, DAB chromogene was used for visualization. Slides were counterstained with Mayer’s hematoxylin, dehydrated and mounted with DPX. Sections were evaluated by means of a light microscope (Leica DM 6000 B) and digitized using a Leica DFC480 digital camera (Leica Microsystems, Wetzlar, Germany).

### Transduction of human embryonic kidney (HEK293) cells and isolation of membrane vesicles

Increased synthesis of human BCRP and MRP3 in HEK293 cells was established using baculoviruses, which were produced using the Bac-to-Bac system (Invitrogen, Breda, The Netherlands) with BacMamVSV-EX-hBCRP (pENTR221-hBCRP; HsCD00044371; Harvard Institute of Proteomics, Harvard Medical School, Boston, MA, USA) and BacMamVSV-EX-hMRP3 (sequence of MRP3 was equal to GenBank accession number NM_003786), as described previously by Wittgen et al. (2011). As a control, the enhanced yellow fluorescent protein (eYFP) was also introduced into the baculoviruses.

Crude membranes of HEK293-BCRP, -MRP3 and -eYFP cells were isolated, resuspended in TS buffer (10 mM Tris-HEPES and 250 mM sucrose, pH 7.4) and membrane vesicles were prepared according to a previously described method (El-Sheikh et al. 2007). Total protein concentration was determined by a Bio-Rad protein assay kit (Bio-Rad Laboratories, Veenendaal, The Netherlands). Crude membrane vesicles were dispensed in aliquots, snap-frozen in liquid nitrogen and stored at −80 °C until further use.

### Western blot analysis

Membrane vesicles were prepared for gel electrophoresis by incubation with Laemmli sample buffer (consisting in 0.5 M Tris–HCl pH 6.8, 8 % w/v sodium dodecyl sulfate (SDS), 40 % w/v glycerol, 0.08 % w/v bromophenol blue and 0.4 M ß-mercapto-ethanol) for 10 min at 65° C. Proteins (15 μg total protein per sample) were separated by SDS-polyacrylamide gel electrophoresis (SDS-PAGE) using a 10 % gel and blotted onto a nitrocellulose membrane using a dry blot system (iBlot; Invitrogen). The membrane was incubated overnight at 4 °C with mouse-anti-hBCRP antibody (BXP-21, 1:200; Kamiya Biomedical) or mouse-anti-hMRP3 antibody (M_3_II-21, 1:200; Abcam, Cambridge, UK). Primary antibodies were detected using fluorescently labeled goat-anti-mouse secondary antibody (IRdye800; 1:10,000 Rockland Immunochemicals, Boyertown, PA, USA). Signals were visualized using the Odyssey imaging system (Li-Cor Biosciences, Lincoln, NE, USA).

### BCRP-mediated estrone sulfate (E_1_S) uptake and inhibition by steroids

Uptake of [^3^H]-E_1_S into HEK293-BCRP and -eYFP membrane vesicles was performed using an assay that was well established in our laboratory (El-Sheikh et al. 2007; Mutsaers et al. 2011; Wittgen et al. 2011). A reaction mix consisting in a TS buffer supplemented with 4 mM ATP/AMP, 10 mM MgCl_2_ and various concentrations of [^3^H]-E_1_S at pH 7.4 was added to 7.5 μg of membrane vesicles (based on total protein content). After an incubation of 60 s at 37 °C to enable ATP-dependent uptake, the reaction was stopped by placing the samples on ice and by addition of an ice-cold TS buffer. The reaction mix was removed and the vesicles were washed by means of a rapid filtration technique using glass fiber filter plates (Millipore, Etten-Leur, The Netherlands). Scintillation fluid was added to the filters and the amount of radioactivity was determined using a scintillation counter (Tri-Carb^®^ 2900TR; Perkin Elmer, Waltham, MA, USA). Reference samples were measured to calculate the amount of transported E_1_S. ATP-dependent transport was calculated by subtracting values measured in the presence of AMP from those measured in the presence of ATP. Net BCRP-mediated E_1_S transport was calculated by subtracting ATP-dependent E_1_S uptake in HEK293-eYFP vesicles from that of HEK293-BCRP vesicles. Linearity of E_1_S uptake in time was determined using 250 nM E_1_S.

The effects of several androgens, estrogens and progestagens (all obtained from Sigma-Aldrich, St. Louis, MO, USA or Steraloids, Newport, RI, USA) on vesicular E_1_S uptake were assessed by performing the above-mentioned transport assay in the presence of 50 μM of the steroid. All steroid hormones were dissolved in ethanol. Maximum ethanol concentrations of 1.3 % v/v were used in uptake experiments and solvent controls were included to exclude non-specific effects. The inhibition by the steroid hormones estradiol, testosterone, progesterone and androstenedione on vesicular E_1_S uptake was studied in more detail. Therefore, the membrane vesicles were incubated in the presence of increasing concentrations of the steroid at three different concentrations of [^3^H]-E_1_S (50, 100 and 250 nM). In studies with androstenedione, the glass fiber filter plate was pre-incubated with 50 mg/ml BSA for 1 h at 37 °C and the reaction mix was supplemented with 0.2 mg/ml BSA, to reduce the background that is caused by adhesion to the filter plate. The sidedness of the membrane vesicles was not determined as ATP-dependent uptake can occur only in inside-out vesicles. The relative inhibition by steroids was expressed as a percentage of maximum uptake.

### Inhibition of MRP3-mediated estradiol glucuronide (E_2_17βG) uptake by steroids

The effects of estradiol, testosterone, progesterone and androstenedione on MRP3 activity was determined by the same method as described above, using E_2_17βG as a substrate. HEK293-eYFP and -MRP3 membrane vesicles were incubated with 80 nM [^3^H]-E_2_17βG with or without the steroids, for 3 min at 37 °C. The washing steps were performed using PVDF filter plates (Millipore). These parameters were adapted from Wittgen et al. (2011). Unlabeled E_2_17βG (100 μM) was used as a positive control for inhibition of MRP3 activity.

### Data analysis

Relative *Bcrp* mRNA concentration was normalized for the cycle threshold (Ct) value of the endogenous reference gene *β-actin* (delta Ct; dCt) and depicted as the reciprocal of dCt (mean ± SEM). Differences in mRNA concentration between male and female organs were assessed by means of a Student’s *t* test, considering *P* < 0.05 significant. Fold differences in *Bcrp* mRNA levels between the organs were calculated using 2^(dCt organ1-dCt organ2)^. Uptake experiments were performed in triplicate in three independent batch isolations. Michaelis–Menten analysis was used to study transport kinetics. Inhibition curves are depicted as mean ± SEM of three independent experiments. Curve fitting was performed by non-linear regression analysis. The mode of inhibition was determined using Dixon’s method combined with linear regression analysis to estimate the inhibitory constant (*K*_i_). A representative of one experiment is depicted. Differences in MRP3-mediated E_2_17βG uptake were assessed by means of a one-way ANOVA test followed by Dunnett’s post hoc test. All analyses were performed with GraphPad Prism software (v.5.02; GraphPad Software, San Diego, CA, USA).

## Results

### *Bcrp* mRNA analysis in murine organs

**Fig. 1:**
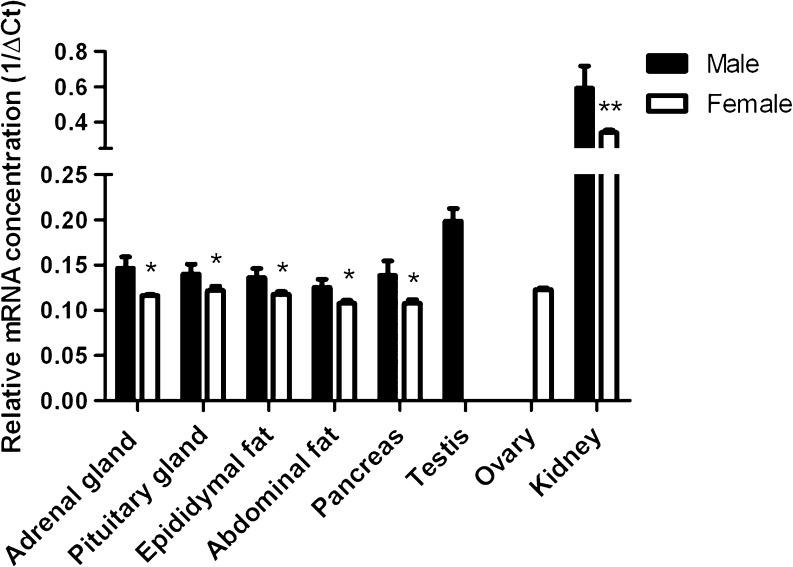
Relative *Bcrp* mRNA concentration in wild-type mouse organs, normalized for the cycle threshold (Ct) value of the endogenous reference gene *β-actin* (delta Ct; dCt). For all experiments, tissues from six mice were individually analyzed, except for the adrenal gland and the pituitary gland. For those, three samples of two pooled organs were analyzed. *Bars* represent the reciprocal of mean dCt ± SEM of independent experiments (*n* = 3–6). *P < 0.05; **P < 0.01 compared to *Bcrp* mRNA concentration in males by Student’s *t* test. There was no difference in *β-actin* mRNA concentration between males and females. The SEM of *β-actin* Ct values was ≤ 0.92

*Bcrp* mRNA levels in murine endocrine organs were determined by means of quantitative PCR (Fig. 1). For comparison, mRNA levels in kidneys were evaluated, which are known to be very high (Huls et al. 2008). The relative *Bcrp* mRNA concentration was normalized to the mRNA concentration of the endogenous reference gene *β-actin*. The variation in cycle threshold (Ct) values of *β-actin* indicated that the QPCR reactions were reproducible (SEM ≤ 0.92). *Bcrp* mRNA was present in all organs tested. The relative mRNA concentration in the adrenal gland, pituitary gland, epididymal and abdominal fat, the pancreas and the ovary was comparable and found to be more than 30 times lower as compared to mRNA levels in the kidney. *Bcrp* mRNA levels in the testis was about four times higher than in the other organs but 9-fold lower as compared to the kidney. Interestingly, mRNA levels of *Bcrp* in male mice were slightly higher than in female mice (*P* < 0.01 for the kidney and *P* < 0.05 for other organs), while there was no difference in *β-actin* mRNA concentration between samples from males and females.

### Localization of Bcrp in murine organs

**Fig. 2:**
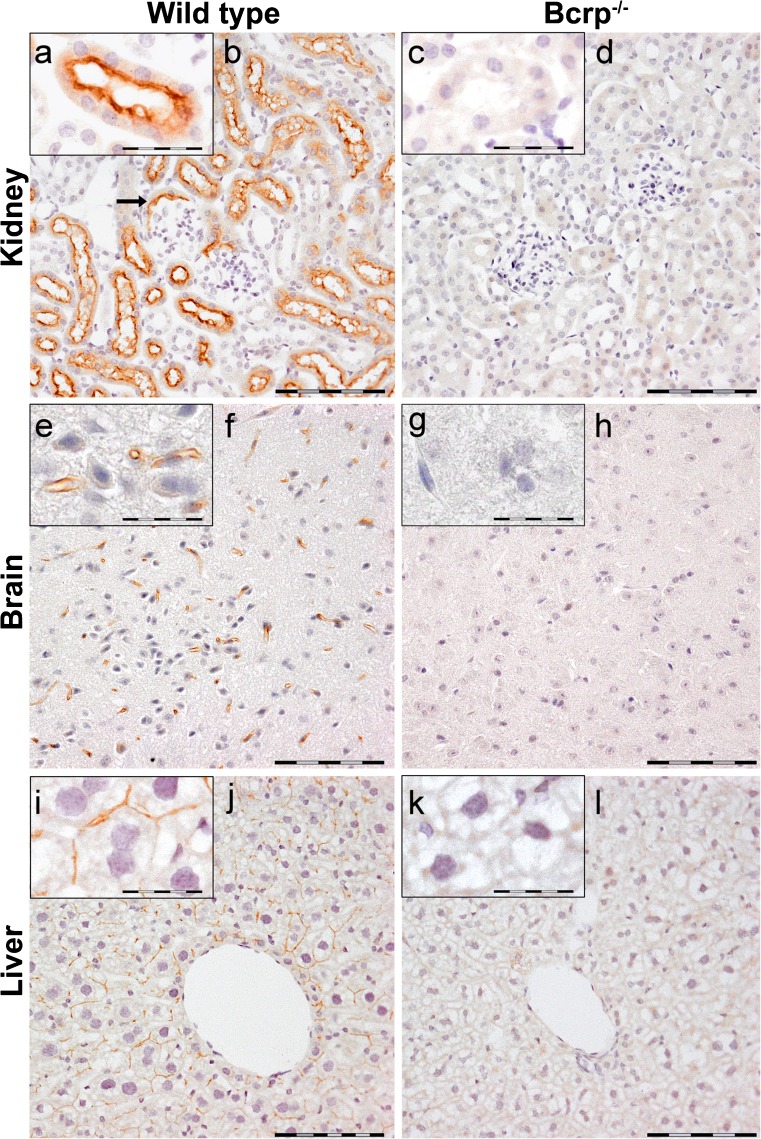
Representative immunohistochemical images of Bcrp distribution in wild-type and Bcrp^−/−^ (negative control) mouse kidney (**a**–**d**), brain tissue (**e**–**h**) and liver (**i**–**l**). Because of an absence of gender difference in localization, only organs from female mice are shown. Perfusion-fixed, paraffin embedded organ sections were incubated with a primary antibody against mouse Bcrp (BXP-9). DAB chromogene staining (*brown*) visualizes Bcrp localization. The sections were counterstained with Mayer’s hematoxylin. *Bars* 100 μm in (**b**, **d**, **f**, **h**, **j**, **l**). **a**, **e**, **i** represent details of Bcrp positivity and corresponding negative controls (**c**, **g**, **k**); *bars* in these *insets* 50 μm. Bcrp was present in the brush border of proximal tubule and in the brush border of Bowman’s capsules (*arrow*) of the kidney and in the capillaries of the brain and liver

**Fig. 3:**
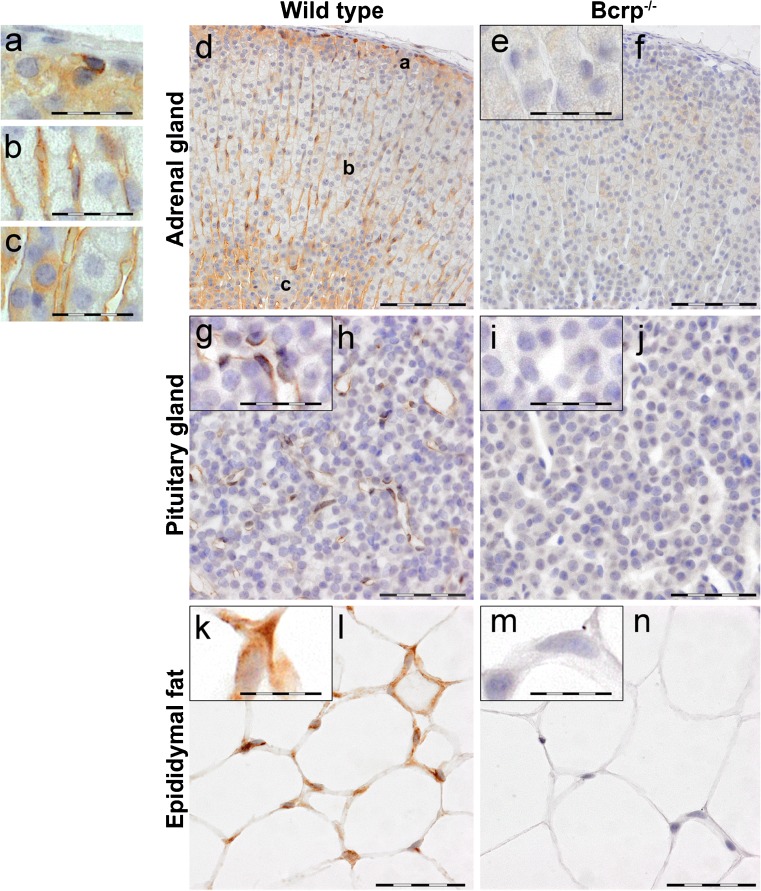
Representative immunohistochemical images of Bcrp distribution in wild-type and Bcrp^−/−^ (negative control) mouse adrenal gland (**a**–**f**), pituitary gland (**g**–**j**) and epididymal fat (**k**–**n**). Because of an absence of gender difference in localization, only organs from female mice are shown. Perfusion-fixed, paraffin embedded organ-sections were incubated with a primary antibody against mouse Bcrp (BXP-9). DAB chromogene staining (*brown*) visualizes Bcrp localization. The sections were counterstained with Mayer’s hematoxylin. *Bars* 100 μm for adrenal gland (**d**, **f**) and 50 μm for pituitary gland (**h**, **j**) and epididymal fat (**l**, **n**). Details of Bcrp positivity in *a* zona glomerulosa, *b* zona fasciculata, *c* zona reticularis of the adrenal gland and corresponding negative controls (**e**) are indicated with a *bar* of 50 μm. **g**, **k** represent details of Bcrp positivity and corresponding negative controls (**i**, **m**) in pituitary gland and epididymal fat; *bars* in these *insets* 25 μm. Bcrp was located in the sinusoidal capillaries in adrenal gland cortex, in the capillaries of the pituitary gland and in adipocytes

**Fig. 4:**
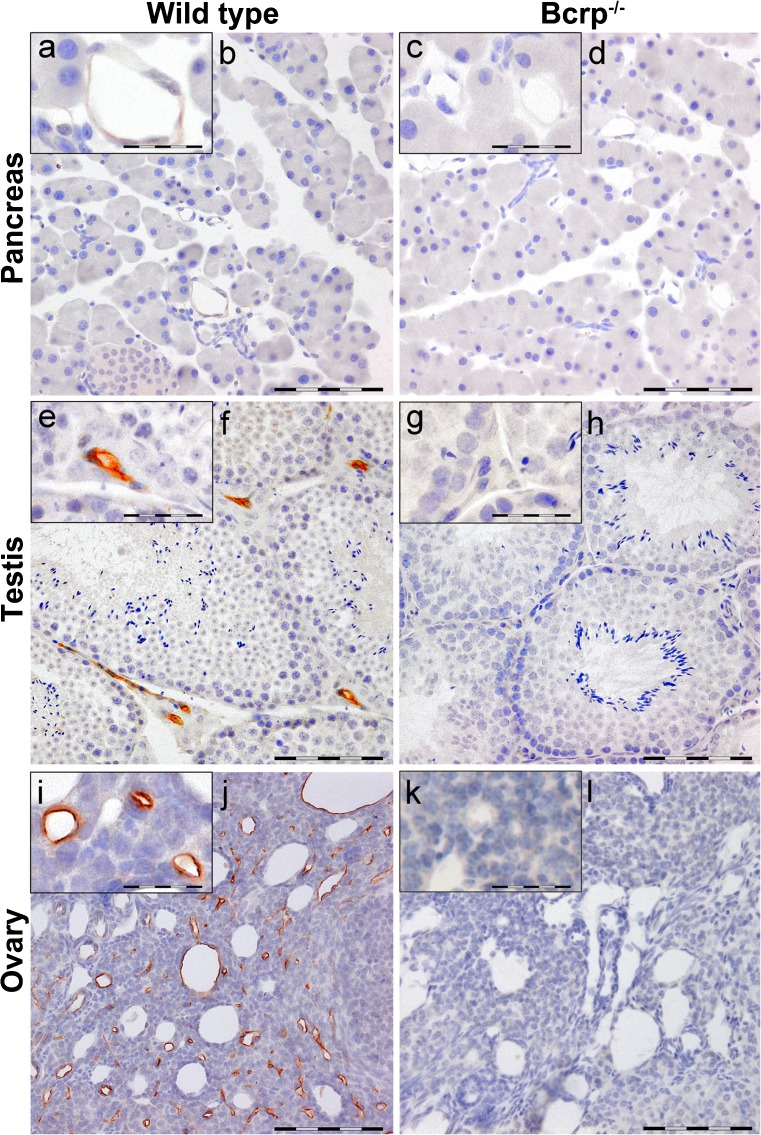
Representative immunohistochemical images of Bcrp distribution in wild-type and Bcrp^−/−^ (negative control) mouse pancreas (female, **a**–**d**), testis (**e**–**h**) and ovary (**i**–**l**). Perfusion-fixed, paraffin embedded organ-sections were incubated with a primary antibody against mouse Bcrp (BXP-9). DAB chromogene staining (*brown*) visualizes Bcrp localization. The sections were counterstained with Mayer’s hematoxylin. *Bars* 100 μm (**b**, **d**, **f**, **h**, **j**, **l**). **a**, **e**, **i** represent details of Bcrp positivity and corresponding negative controls (**c**, **g**, **k**); *bars* in these *insets* 50 μm. Blood vessels in the testis and ovary showed distinct Bcrp positivity, whereas in the pancreas, they showed very subtle Bcrp staining

Immunohistochemical analysis revealed localization of Bcrp protein in numerous murine organs. Organ sections of Bcrp^−/−^ mice were used to determine nonspecific staining. Both male and female organ sections were evaluated but no differences in localization of the protein between male and female were found (data not shown). Therefore, only organs from female mice are shown here, except for the testis. Fig. 2 depicts Bcrp localization in organs with a barrier function, viz. kidney, brain and liver. In the kidney, Bcrp staining was most prominently observed in the proximal tubular lumen, the brush border (Fig. 2a). Also, Bcrp was localized in Bowman’s capsule brush border, which is present in murine nephrons. The distal tubular lumen did not contain Bcrp. No Bcrp staining was found in the negative control sections (Bcrp^−/−^; Fig. 2c, d, g, h, k, l). Furthermore, Bcrp was localized in the capillaries of the brain and liver (Fig. 2e, f, and i, j, respectively). Besides presence of Bcrp in organs with a barrier function, the protein was found in endocrine organs (Figs. 3 and 4). Bcrp was clearly localized in the sinusoidal capillaries in the adrenal gland cortex (Fig. 3a–d). In addition to this, some cells showed intracellular Bcrp staining in the zona glomerulosa (Fig. 3a) and zona reticularis (Fig. 3c) but intracellular Bcrp staining was not found in either the zona fasciculata (Fig. 3b) or the medulla of the adrenal gland (Fig. 3d). In the capillaries of the anterior pituitary gland, Bcrp was localized at the apical surface of the endothelial cells (Fig. 3g, h). The hormone producing cells of the pituitary gland showed no Bcrp-specific staining. The adipocytes in both epididymal and abdominal fat pads showed Bcrp positivity, which was absent in Bcrp^−/−^ sections (Fig. 3k, l). No differences in localization of the pump were found between epididymal and abdominal fat pads and therefore only micrographs of epididymal fat pads are shown in Fig. 3k, l. Bcrp was not located in the endocrine and exocrine cells of the pancreas (Fig. 4a, b) but endothelial cells lining the lumen of the capillaries in the pancreas showed a very subtle Bcrp staining (Fig. 4a), when comparing wild-type organs with the negative control organs. Blood vessels of the testis, located between the seminiferous tubules, were Bcrp-positive (Fig. 4e, f). On the other hand, the interstitial cells of Leydig and the Sertoli cells, which were located within the seminiferous tubules, did not show Bcrp staining. Furthermore, the transport protein was localized in endothelial cells lining the lumen of ovarian blood vessels (Fig. 4i, j). Interestingly, not all vessels were Bcrp-positive but Bcrp was present in all small capillaries.

### Presence and activity of BCRP in isolated membrane vesicles

**Fig. 5:**
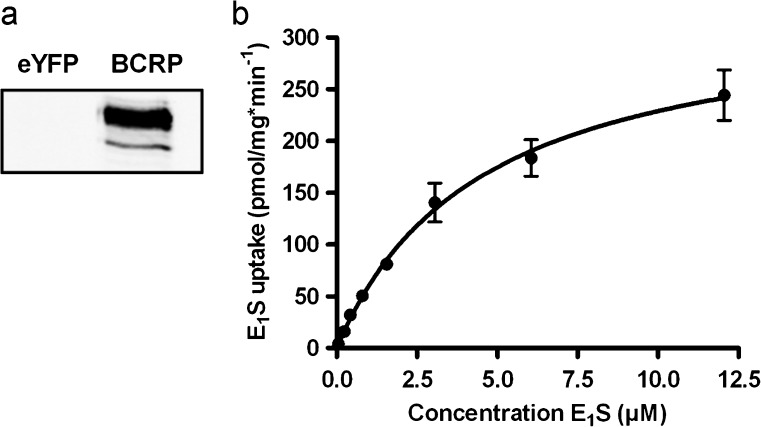
**a** Western blot analysis of HEK293-eYFP- and -BCRP membrane vesicles. BCRP was detected using mouse-anti-BCRP antibody (BXP-21). A Michaelis–Menten plot (**b**) revealed net BCRP-mediated uptake of E_1_S. Vesicles were incubated with increasing concentrations of [^3^H]-E_1_S for 60 s at 37 °C in the presence of AMP or ATP. Net ATP-dependent uptake was calculated by subtraction of AMP values. Control (eYFP) vesicles showed maximum uptake of 6 pmol/mg min^−1^. *Graph* represents means ± SEM of triplicate measurements in a representative experiment

Increased synthesis of human BCRP in HEK293 cells was established by transduction with baculovirus containing the recombinant bacmide DNA. Western blot analysis performed on isolated membrane vesicles demonstrated that the transduction was successful, as indicated by the band at ~75 kD (Fig. 5a). The negative control, consisting in membrane vesicles isolated from HEK293-eYFP cells, showed no presence of BCRP. Transport activity was determined by incubating the membrane vesicles with [^3^H]-E_1_S at 37 °C. Time-dependent uptake showed linearity up to 120 s (data not shown). Michaelis–Menten analysis (Fig. 5b) revealed a Km of 4.5 ± 0.4 μM and a Vmax of 332 ± 12 pmol/mg min^−1^, after incubating the vesicles for 60 s at 37 °C. In control (eYFP) vesicles, a maximum uptake rate of 6 pmol/mg min^−1^ was found.

### Inhibition of transport activity of BCRP and MRP3 by steroids

**Fig. 6:**
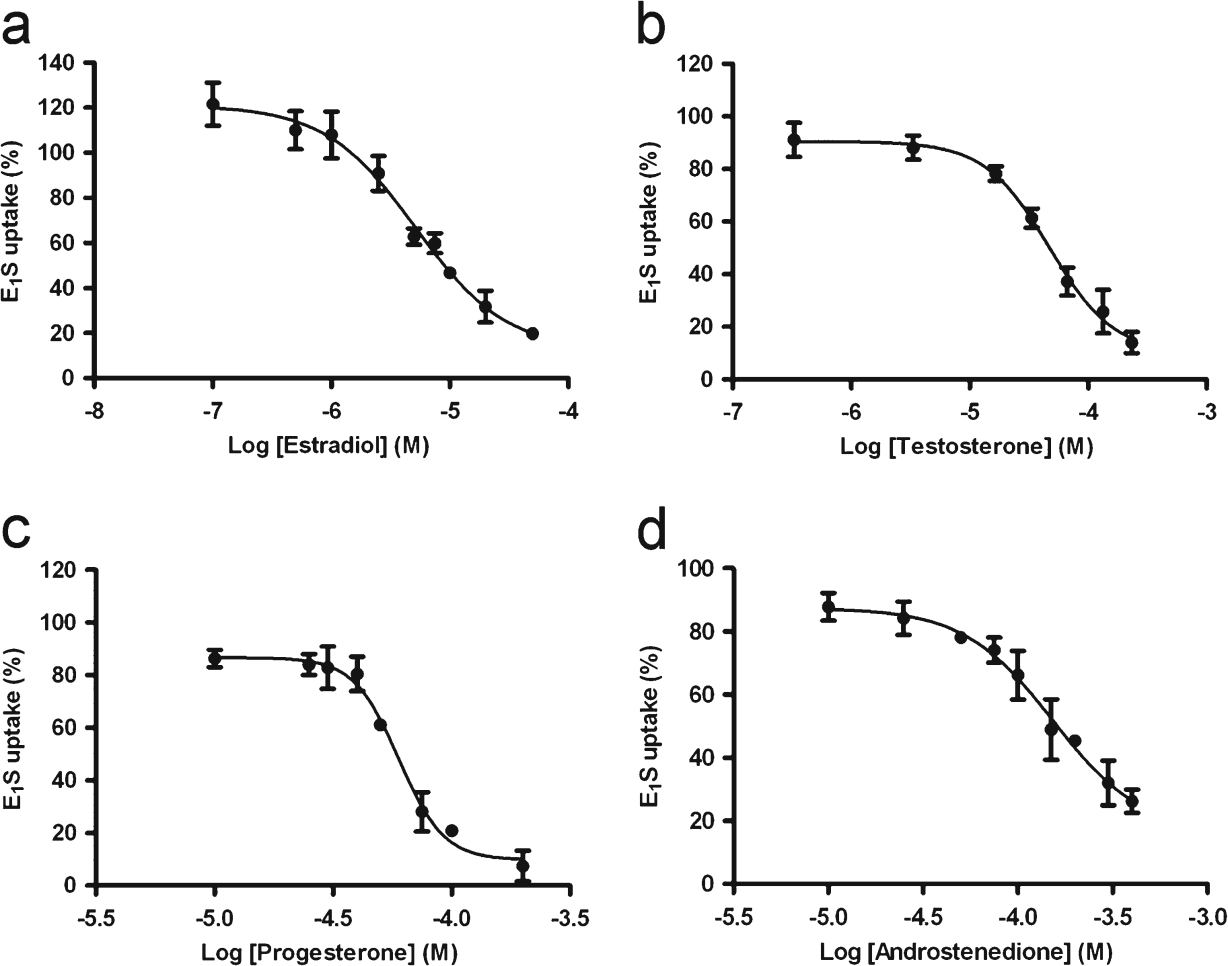
Concentration-dependent inhibition of net BCRP-mediated E_1_S uptake by estradiol (**a**), testosterone (**b**), progesterone (**c**) and androstenedione (**d**). Vesicles were incubated with 250 nM [^3^H]-E_1_S and increasing concentrations of the steroid, for 60 s at 37 °C in the presence of AMP or ATP. AMP values were subtracted from ATP values. Net BCRP-mediated E_1_S uptake was calculated by subtraction of corresponding eYFP values and expressed as a percentage of maximum uptake. Curve fitting was performed by non-linear regression analysis. *Graphs* represent means ± SEM of three independent experiments

The effects of several androgens, estrogens and progestagens on BCRP-mediated E_1_S uptake was assessed by incubating the membrane vesicles with [^3^H]-E_1_S in the presence of 50 μM of the steroid for 60 s at 37 °C. A substrate concentration of 250 nM E_1_S was used (Fig. 5b), which was well below the Km of E_1_S uptake to ascertain initial uptake kinetics. Estradiol, testosterone, progesterone and androstenedione inhibited the efflux pump most potently (Table 1) and were, therefore, selected to be studied in more detail. Concentration-dependent inhibition of BCRP-mediated uptake of E_1_S was found for all four steroids (Fig. 6a–d). Progesterone was able to inhibit BCRP activity for >90 % within the concentration-range tested. Estradiol, testosterone and androstenedione inhibited the efflux pump for 80, 86 and 70 %, respectively, at the highest possible concentrations tested. We were not able to assess whether the steroids were able to fully inhibit the efflux pump using our vesicular transport system because this was limited by the maximum solubility of the steroids in ethanol.

**Table 1:** Effect of steroids (50 μM) on BCRP-mediated vesicular E_1_S uptake

Steroid	Vesicular E_1_S uptake (%)
Cholesterol	71.9 ± 5.2
Pregnenolone	ND
17α-hydroxypregnonolone	77.2 ± 17.1
Dehydroepiandrosterone	58.7 ± 7.8
Androstenediol	70.2 ± 18.7
Progesterone	28.7 ± 14.4^a^
17α-hydroxyprogesterone	108.0 ± 18.7
Androstenedione	42.4 ± 9.4^a^
Testosterone	20.1 ± 12.2^a^
Dihydrotestosterone	48.4 ± 24.6
Estrone	ND
Estradiol	40.4 ± 8.4^a^
Estriol	53.1 ± 22.5

Values are shown as mean ± SEM of one experiment performed in triplicate. Vesicular E_1_S uptake is expressed as a percentage of maximum uptake after incubation with 250 nM [^3^H]-E_1_S with or without 50 μM of the steroid for 60 s at 37 °C

*ND* Not determined

^a^The most potent inhibitors of BCRP-mediated E_1_S uptake were selected to be studied in more detail

**Fig. 7:**
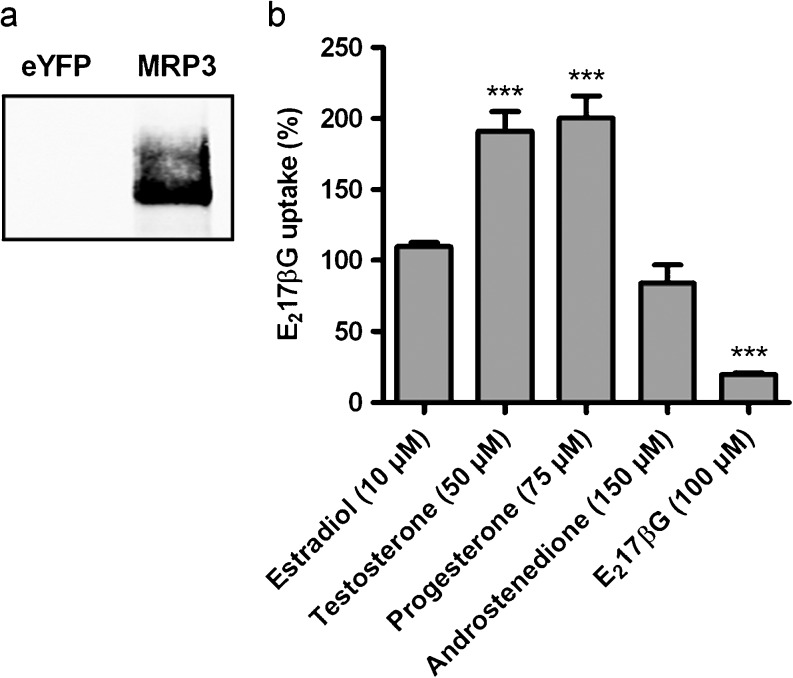
**a** Western blot analysis of HEK293-eYFP- and -MRP3 membrane vesicles. MRP3 was detected using mouse-anti-MRP3 antibody (M_3_II-21). **b** Effects of estradiol, testosterone, progesterone and androstenedione on MRP3 activity. Membrane vesicles were incubated with 80 nM [^3^H]-E_2_17βG (substrate) and indicated concentrations of the steroids, for 3 min at 37 °C. The effect of the steroids on net MRP3-mediated E_2_17βG uptake is expressed as a percentage of uptake in negative control samples. *Bars* represent means ± SEM of three independent experiments. Differences in MRP3-mediated E_2_17βG uptake were assessed by means of a one-way ANOVA test followed by Dunnett’s post hoc test

Because of their lipophilicity, steroids move readily through the plasma membrane and potentially might interfere with other membrane transporters. To exclude the possibility that the inhibitory effects on BCRP activity resulted from such a nonspecific phenomenon, the steroids were tested for their inhibitory effects on the activity of another ABC transporter, viz. MRP3. Unlike substrates for other ABC transporters, such as MRP1, MRP2 and P-glycoprotein, MRP3 substrates do not overlap with the ones known to be transported by BCRP. Because of this, we did not expect steroids to interact with MRP3. Increased synthesis of human MRP3 in HEK293 membranes was confirmed using western blot analysis (Fig. 7a). Concentrations of the steroids that were able to inhibit BCRP activity for more than 50 % were used to determine their effects on MRP3 activity, using E_2_17βG as a substrate. Under these conditions, none of the steroids inhibited MRP3-mediated [^3^H]-E_2_17βG uptake (Fig. 7b). Instead, testosterone and progesterone even stimulated MRP3 activity. Moreover, the positive control, unlabeled E_2_17βG (100 μM), did inhibit [^3^H]-E_2_17βG uptake significantly, to 19.4 ± 0.6 % of maximum uptake (*P* < 0.001).

### Dixon plot analysis

**Fig. 8:**
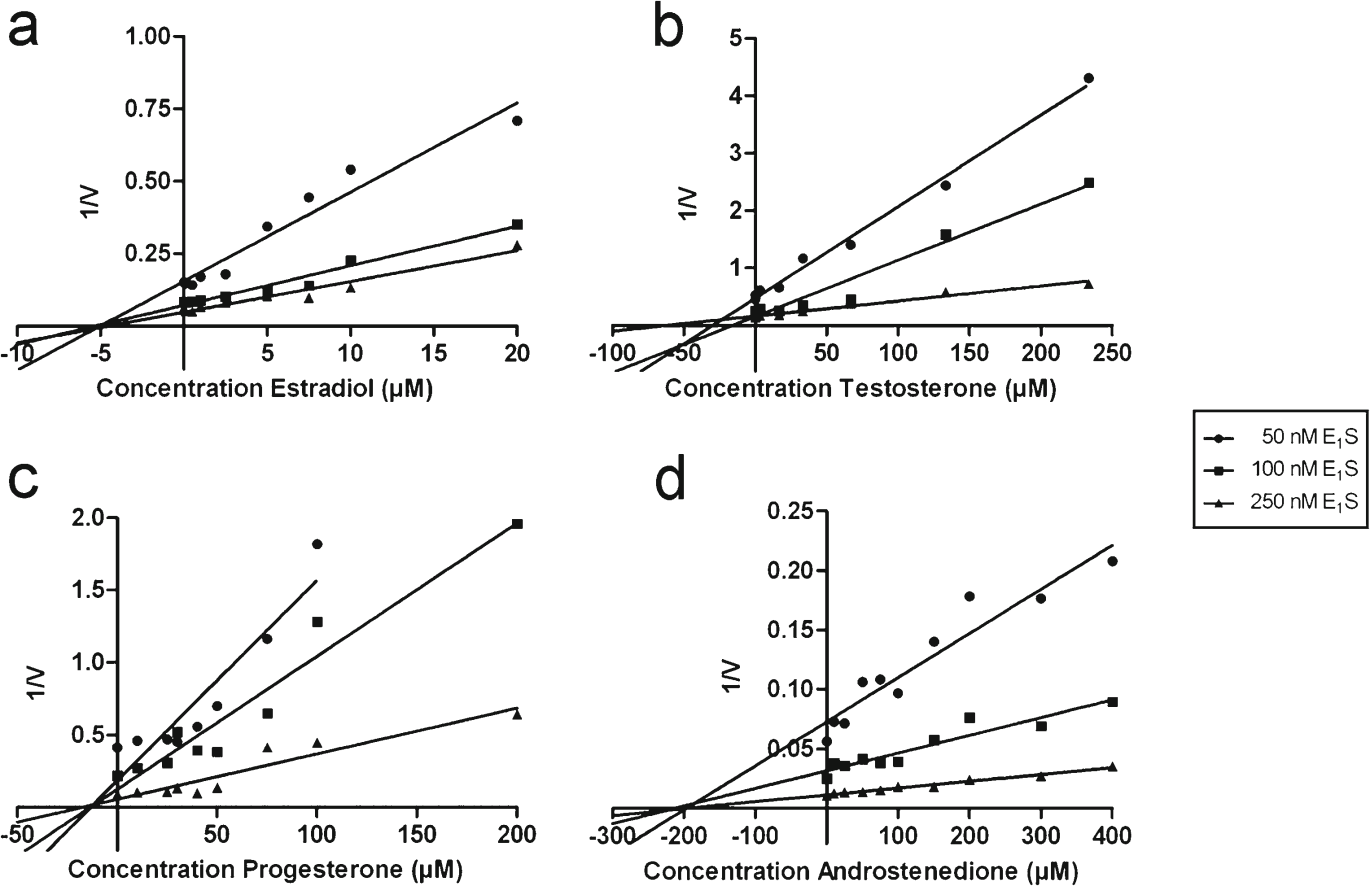
Dixon plots of inhibitory effects of estradiol (**a**), testosterone (**b**), progesterone (**c**) and androstenedione (**d**) on BCRP-mediated E_1_S uptake. Membrane vesicles were incubated with indicated concentrations of [^3^H]-E_1_S and increasing concentrations of the steroid. The reciprocal of transport velocity (1/V) was calculated and the mode of inhibition was determined using Dixon’s method combined with linear regression analysis to estimate the inhibitory constant (*K*_i_) values; **a** 5.0 ± 0.2 μM; **b** 36 ± 14 μM; **c** 14.7 ± 1.3 μM; **d** 217 ± 13 μM). A representative of one experiment, out of three independent measurements, is depicted

To determine the mode of interaction, we measured concentration-dependent effects of the steroids on BCRP activity at three different E_1_S concentrations. Fig. 8a–d depicts Dixon plots of the data, which were analyzed by linear regression. The three lines representing steroid inhibition at different E_1_S concentrations of the four steroids all intersected at the *x*-axis, indicating a non-competitive inhibitory effect. The apparent inhibitory constant (*K*i) values were 5.0 ± 0.2 μM for estradiol, 36 ± 14 μM for testosterone, 14.7 ± 1.3 μM for progesterone and 217 ± 13 μM for androstenedione.

## Discussion

This study reports a clear overview of Bcrp presence in several murine organs as well as its cellular localization in these tissues. In addition to adrenal gland, pancreas and testis, we show for the first time localization of Bcrp in ovary, pituitary gland and adipose tissues. Moreover, we report an efficient inhibitory effect of steroid hormones on BCRP function. Estradiol, testosterone, progesterone and androstenedione attenuated BCRP-mediated E_1_S transport in a non-competitive manner, which implies an allosteric inhibition of BCRP function by these steroids.

Presence of BCRP in various tissues with a barrier function, including placenta, prostate, small intestine, brain, colon, liver, mammary gland and kidney has been shown previously (Maliepaard et al. 2001; Doyle and Ross 2003; Bart et al. 2004; Fetsch et al. 2006; Huls et al. 2008). Investigations on the presence of the efflux pump in endocrine organs has received much less attention and localization of the protein has hardly been described. We assessed Bcrp on both mRNA level and protein presence in endocrine organs. In addition, endocrine organs from Bcrp^−/−^ mice were used for comparison, allowing discrimination between background and Bcrp-specific staining. In accordance with the findings of Langmann et al. (2003), who quantified *Bcrp* mRNA in various human tissues, we found *Bcrp* mRNA in mouse kidney, testis and adrenal gland. While our results show highest mRNA levels in the kidney, Langmann et al. described highest mRNA levels in the testis. This could be attributed to interspecies differences. We have previously described that Bcrp mRNA levels were much higher in mouse kidneys compared to rat and human kidneys (Huls et al. 2008). Additionally, our results revealed that, for all organs tested, mRNA concentrations of *Bcrp* in male mice were significantly higher than in females, which is in agreement with previously published findings. Despite the clear gender differences in *Bcrp* mRNA levels, there were no differences in localization of the protein between male and female organs (data not shown). Tanaka et al. (2005) described male-predominant *Bcrp* gene expression in rat kidney and mouse liver. Moreover, Merino et al. (2005) found differences in the disposition of the BCRP substrate, nitrofurantoin, between male and female mice, with higher plasma levels in females. They also reported higher hepatic protein levels of human BCRP in men compared to women, determined by western blot analysis using crude membrane fractions from human liver. The precise role of the efflux pump in males versus females was beyond the scope of the present study and further research should elucidate the consequences of this gender difference.

In accordance with previous findings (Huls et al. 2008), immunohistochemical analysis revealed that Bcrp was prominently located in the brush borders of proximal tubule epithelium of the murine kidney. Furthermore, Bcrp was found to be present in the adrenal gland, pituitary gland, testis and ovary, where it was located predominantly in endothelial cells lining blood vessel walls. In agreement, Maliepaard et al. (2001) reported Bcrp localization in venous and capillary endothelial cells of almost all tissues.

In addition to the arterioles in the adrenal gland cortex, Bcrp was found to be present in the cells lining the zona glomerulosa and zona reticularis, which secrete mineralocorticosteroids and androgens, respectively. The latter is in agreement with the findings of Fetsch et al. (2006). For the pituitary gland, we are the first to reveal Bcrp localization in the capillaries. Bcrp presence in blood vessels was also found in the pancreas, though it was low compared to the other organs studied. The presence of Bcrp in the pancreas has been described previously but mainly in pancreatic progenitor cells (Mato et al. 2009; Zhang et al. 2005). In contrast to Fetsch et al. (2006), who reported Bcrp localization in the islet and acinar cells, we did not find Bcrp in the endocrine and exocrine cells of the pancreas.

In ovary, Bcrp was clearly present in capillaries and some larger blood vessels and the capillaries also showed positive staining in the testis. The fact that not all vessels in ovary tissue were Bcrp-positive might be characteristic for the dynamic environment of the estrous cycle, where there is a constant regeneration and degradation of capillaries. The degrading capillaries lose their endothelial function and thereby endothelial protein is decreased. Tanaka et al. (2005) reported Bcrp in ovary and testis of mice. In accordance with our results, they demonstrated that Bcrp protein levels were higher in testis than in ovary. Enokizono et al. (2007) did not find evidence for Bcrp activity when evaluating genistein accumulation in the ovaries of wild-type and Bcrp^−/−^ mice but they did find Bcrp activity in murine testis. It was reported that the interstitial cells of Leydig (Fetsch et al. 2006) and spermatogonial cells (Lassalle et al. 2004) of the testis localized Bcrp. Conversely, our findings could not endorse these reports. However, the presence of Bcrp in the lumina of testicular blood vessels was evident (Bart et al. 2004).

In both epididymal and abdominal fat pads, we found Bcrp to be present in mature adipocytes, which has not previously been described. Adipocytes are metabolically active cells that play a key role in the control of the body's energy balance by the secretion of resistin, adiponectin and leptin. Moreover, adipocytes are involved in steroid hormone regulation since they highly express aromatase (*CYP19A1*), which is responsible for a key step in the biosynthesis of estrogens (Raven et al. 2006). Therefore, it will be interesting to further unravel the role of Bcrp in adipocytes.

Another important endocrine organ, which we did not evaluate, is the mammary gland. Steroids greatly influence the growth and function of the mammary gland. It is, therefore, interesting to study the presence and function of Bcrp in this organ. In 2005, Jonker et al. (2005) published the localization of BCRP/Bcrp in breast tissue of mice, cows and humans. They clearly showed that the transporter was localized in mouse mammary gland epithelium during late pregnancy and in lactating mice but presence of the protein was negligible in the mammary gland of nonpregnant and nonlactating mice. It would be interesting to study the mechanism by which Bcrp synthesis and function is influenced during pregnancy and lactation.

In all the organs we analyzed, Bcrp was principally found in the endothelial layer of sinusoids, capillaries and veins. The fact that Bcrp was located in the canalicular membrane of the liver indicates that the efflux pump may be involved in excretion processes in the liver, similar to other ABC transporters, such as P-gp and MRP2 (Maliepaard et al. 2001). In the brain and the testis, the endothelial cells form tight junctions, creating a blood–tissue barrier, preventing substances from freely entering the tissue. Here, efflux transporters, such as Bcrp, are thought to protect the brain and developing sperm cells against toxic agents. In other organs, normal endothelium consists in loosely connected endothelial cells and is known to be quite permeable for several substances. Nevertheless, the fact that Bcrp is so clearly present in these endothelial cells and that it is able to transport substances against a concentration gradient indicates a contribution of the efflux pump to transport compounds across the endothelium. Although, the endocrine organs, obviously, have a significant secretory function, we did not find Bcrp localized in endocrine and exocrine cells, except for adipocytes and the adrenal gland. Hence, the efflux pump may contribute to the transport of steroids, or their derivates, into the blood stream.

Using Dixon’s method, we proved indirectly that estradiol, testosterone, progesterone and androstenedione are efficient inhibitors but likely not transported by BCRP via the same binding site as E_1_S. The question whether steroids are substrates for BCRP has been discussed before and contradictory findings have been reported (Huss et al. 2005; Imai et al. 2003; Janvilisri et al. 2003). Most studies support the conclusion that steroids interact but are not substrates for BCRP, which is in agreement with the present findings. Nevertheless, we cannot completely rule out the possibility of steroids being transported by BCRP. The idea that there is no need for steroids to be transported actively over the plasma membrane because of their high lipophilicity is outdated, since it has been described that steroids are substrates for other ABC transporters (van Kalken et al. 1993; Drobnik et al. 2001; Wolf and Horwitz 1992; Dikkers and Tietge 2010). Besides, steroids are derived from cholesterol, which itself is transported by other members of the ABCG subfamily (Graf et al. 2003; Schmitz et al. 2001). We have undertaken studies to measure steroid uptake in HEK293-BCRP membrane vesicles but unreliable results were obtained due to the large passive permeability of these lipophilic compounds.

To exclude the possibility that the inhibitory effects of the steroids on BCRP activity were nonspecific, their effects on the activity of MRP3 was tested. None of the steroids inhibited MRP3-mediated E_2_17βG uptake. Interestingly, however, testosterone and progesterone stimulated this uptake. Stimulation of MRP3 has been described before, although the mechanism explaining these complex stimulatory effects has not yet been elucidated (Akita et al. 2002; Chu et al. 2004; Wittgen et al. 2011). The fact that MRP3 function was not inhibited by the steroids indicates that the inhibition of BCRP activity by the steroids was a transporter-specific effect.

Estradiol, testosterone, progesterone and androstenedione inhibited BCRP activity by more than 70 %. However, the concentrations needed to cause this inhibitory effect were rather high compared to plasma levels. These concentrations are indicative of the affinity of the steroids for BCRP compared to the affinity of the substrate (E_1_S) for BCRP, which is known to be very high. When the affinity of the steroids is much lower, then the concentrations needed to cause an effect are obviously much higher. Furthermore, the steroids themselves are very lipophilic and are therefore prone to stick to plastic tubes and/or wells, which might result in an over-estimation of the actual concentration. This problem was acknowledged earlier by Tanneberger et al. (2010). To predict the clinical relevance of the interaction between steroids and BCRP, knowledge on intracellular steroid concentrations is important. Plasma steroid levels are not relevant for predicting their effects on a membrane transporter that transports molecules from the inside to the outside of the cell. Intracellular levels are, so far, unknown and difficult to determine. One can imagine that intracellular concentrations in organs responsible for the production of hormones may be much higher than plasma levels and, thereby, could potentially influence BCRP function in vivo.

Not only BCRP function is altered by steroids, as shown in our results but membranal localization of BCRP is also highly influenced by sex steroids. Imai et al. (2005) found that estrogens post-transcriptionally downregulated BCRP in estrogen-responsive cancer cells. They found estradiol-mediated reduction in BCRP protein in MCF-7 cells but not on the mRNA level. This downregulation was counteracted by gene silencing of estrogen receptor-α (ERα), indicating that ERα is necessary for the suppression of BCRP protein. In agreement, Hartz et al. (2010) described that estradiol signals through ERβ and ERα to initiate Bcrp internalization and acts via ERβ to stimulate proteosomal degradation of Bcrp in murine brain capillaries. In contrast, Ee et al. (2004) found that estradiol enhanced *Bcrp* mRNA levels in cells stably expressing *ERα*, at similar estradiol concentrations. Ovariectomy and castration of mice and rats, solely or in combination with sex steroid treatment, revealed regulation of *Bcrp* mRNA by estradiol and testosterone (Tanaka et al*.* 2005). In human placental BeWo cells, progesterone and estradiol significantly increased and decreased *BCRP* mRNA levels, respectively (Wang et al. 2006). Estradiol by itself likely downregulated BCRP through an estrogen receptor (ER), while progesterone alone upregulated BCRP via a mechanism other than progesterone receptor (PR).

In conclusion, we found Bcrp to be present in numerous murine endocrine organs, including ovary, pituitary gland and adipose tissues. Furthermore, the efficient inhibition of BCRP-mediated transport by estradiol, testosterone, progesterone and androstenedione implies a clear interaction between the steroids and the efflux pump. Together, our results support the speculation that BCRP has a role in steroid hormone regulation. Further research will address this hypothesis.
